# Development and Usability Testing of the Mobile Childhood Asthma Management Program (mCHAMP) App: Sequential Mixed Methods Study

**DOI:** 10.2196/71681

**Published:** 2026-03-20

**Authors:** Robert J Lucero, Kristen Shear, Andrea Fidler, David Fedele, Yunpeng Xia, David Janicke

**Affiliations:** 1 Chicano Studies Research Center School of Nursing University of California, Los Angeles Los Angeles, CA United States; 2 College of Nursing University of Florida Gainesville, FL United States; 3 Center for Nursing Science and Clinical Inquiry San Antonio Military Medical Center San Antonio, TX United States; 4 Department of Child and Adolescent Psychiatry and Behavioral Sciences Children's Hospital of Philadelphia Philadelphia, PA United States; 5 Nemours Children Research Nemours Children's Health System Jacksonville, FL United States; 6 School of Nursing University of Alabama at Birmingham Birmingham, AL United States; 7 Department of Clinical and Health Psychology University of Florida Gainesville, FL United States

**Keywords:** health promotion, self-management, mobile health, mHealth, asthma, obesity, pediatrics, usability, family, family caregiver, ambulatory nursing

## Abstract

**Background:**

More than 6 million children in the United States have asthma, and more than 20% are clinically obese. Youth with asthma and obesity are susceptible to poor health outcomes, including greater asthma symptom severity and hospitalizations, reduced physical activity, and poorer quality of life. Mobile health technologies can increase access to chronic disease self-management interventions, and family members can be powerful influencers given their substantial control over a child’s behavior and home environment.

**Objective:**

The Mobile Childhood Asthma Management Program (mCHAMP) app is based on the in-person Childhood Asthma Management Program (CHAMP) behavioral family intervention pilot trial of school-aged children with asthma and obesity. In this study, we translated the CHAMP content into digital content and conducted summative testing to measure the usability, learnability, and efficiency of the mCHAMP app.

**Methods:**

We applied a sequential mixed methods approach. The mCHAMP app targeted adult caregivers of children living with asthma and obesity aged 6 to 12 years. A consumer-centered approach was used to guide the identification of user requirements and conduct of summative usability testing. While the mCHAMP app is primarily caregiver facing, it is intended to connect caregivers with registered nurse (RN) interventionists. Therefore, we sought feedback from RNs as key stakeholders.

**Results:**

Caregivers (n=10) were female (n=10, 100%) and mostly African American (n=8, 80%), and half (n=5, 50%) had an annual household income of <US $25,000; most of their children (n=6, 60%) were in the 99th BMI percentile. Post-Study eHealth Usability Questionnaire scores indicated high overall satisfaction, usefulness, and quality of the mCHAMP app. Most caregivers (n=7, 70%) were able to complete all 15 tasks across the 6 modules with 2 or fewer hints. The average total time to complete all tasks was 17 (SD 3.9; range 11.4-24.1) minutes. Most caregivers (n=7, 70%) wanted information in static form but also preferred alternatives (eg, audio or video) to support flexibility with consuming the content. Caregivers expressed the need for more child-facing content as well as tailored decision support related to diet and exercise. RNs (n=5) strongly endorsed their role and use of the mCHAMP app to promote self-management among caregivers of children with obesity and asthma. They noted the importance of integrating the mCHAMP app into a local electronic health record and existing workflows.

**Conclusions:**

Caregivers expressed a desire for an intervention that was easy to use and could integrate into their busy family lives. We met this expectation based on the usability, learnability, and efficiency results of our study. The mCHAMP app has the potential to increase self-management for parents and pediatric patients with asthma with multimorbidity, which could improve patient and health system outcomes. The use of mCHAMP may also enable novel clinical outcome studies based on patient-reported data from the app.

**International Registered Report Identifier (IRRID):**

RR2-10.2196/13549

## Introduction

### Background

More than 6 million children in the United States have asthma, and more than 20% are clinically obese [1]. Asthma and obesity are leading causes of morbidity, reduced quality of life, and health care costs [2]. Youth with asthma and obesity are especially susceptible to poor health outcomes, including greater asthma symptom severity and hospitalizations, reduced physical activity, and poorer quality of life [3-5]. Children with asthma and obesity can improve their overall health and quality of life when families engage in chronic disease self-management behaviors, including self-monitoring (eg, symptoms and diet), setting and achieving goals (eg, increasing physical activity), modifying the home environment (eg, trigger reduction and healthy food options), and collaborating with a health care provider [6-8].

Caregivers (ie, family members) can be powerful influencers of school-aged children (aged 6-12 years) during this developmental stage given their substantial direct and indirect control over a child’s behavior and home environment [9-11]. However, family caregivers often have limited chronic disease self-management knowledge and struggle themselves to adhere to healthy behaviors [12-18]. There is a critical need for interventions that target family caregiver self-management behaviors during school age for children with asthma and obesity to optimize health and model self-management behaviors that can prevent downstream morbidity in adolescence and beyond [19].

Behavioral family lifestyle interventions (BFIs) can reinforce family caregivers’ self-management skills, resulting in health benefits for their children [20]. BFIs are informed by social cognitive theory, which posits that caregivers need knowledge and skills to engage in health-promoting behaviors [21,22]. BFIs target family caregivers as primary agents of change for health promotion. Caregivers learn child behavior management skills (eg, modeling and contingency management) to help them motivate and support their children in making healthy choices. BFIs can result in family caregivers developing self-management skills and self-efficacy to regulate their children’s behaviors and creating a healthy environment [23,24].

BFIs are the most effective interventions for children with obesity [25,26]. Caregiver self-management skills promoted in BFIs are also critical for successful asthma management [27-29]. However, caregivers report numerous social determinant of health–related barriers (eg, access to health care services and health IT) to attending in-person sessions that limit self-management skill acquisition, behavior change, and benefits to their children [30-32].

Mobile health (mHealth) technologies can increase access to chronic disease self-management interventions. This is especially important given the ubiquity of smartphone ownership across racial and ethnic groups and socioeconomic strata [33,34]. Consumers perceive the usability and acceptability of mHealth interventions as very high. mHealth functionality can be multimodal (ie, text, audio, and visual) to accommodate various literacy levels. mHealth-supported interventions can enable flexibility in accessing and completing intervention components to improve intervention uptake and reduce the time commitment of consumers [35,36]. mHealth-enabled BFIs can reduce barriers for family caregivers in learning and role-modeling self-management skills to improve their children’s asthma and obesity [30-32]. In addition, mHealth self-management interventions show promise in improving youth asthma and weight outcomes [37-40]. Individual tailoring to the caregiver via ongoing support from a health care provider can facilitate intervention engagement [39,41,42]. This is particularly important to caregivers of children with asthma and obesity as this multimorbidity adds significant complexity to disease management for caregivers [20]. Caregivers of children with asthma and obesity conveyed in our pilot work that they would value ongoing contact with a registered nurse (RN) as support for self-management. RNs have the requisite knowledge and skills to educate, motivate, and successfully assist families in using self-management skills to facilitate health behavior change, making them the ideal health professionals to support an mHealth-enabled BFI for caregivers of school-aged children with asthma and obesity [43,44].

### Objectives

The Mobile Childhood Asthma Management Program (mCHAMP) is based on lessons learned from our in-person Childhood Asthma Management Program (CHAMP) pilot trial, a BFI with families of school-aged children (6-12 years) with asthma and obesity [45]. The CHAMP intervention included 16 sessions over 3 months and encompassed topics such as collaborative ways for caregivers and children to establish healthier eating and exercise habits, healthy eating on a budget, goal setting, and progress tracking. In this study, we modified CHAMP into mCHAMP in 2 phases. Following the translation of CHAMP content, summative testing was used to measure usability, learnability, and efficiency. The focus of this paper is the summative usability testing of the mCHAMP app. However, a brief discussion of system development and formative testing is included for context.

## Methods

**Figure 1:**
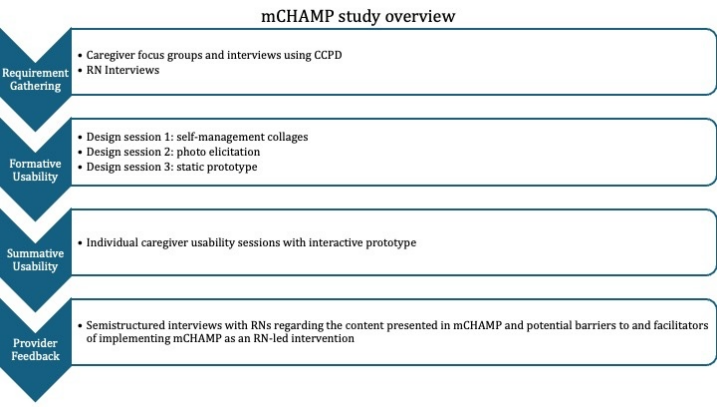
Overview of the study design. CCPD: consumer-centered participatory design; mCHAMP: Mobile Childhood Asthma Management Program; RN: registered nurse.

An overview of this sequential mixed methods study is shown in Figure 1. Additional study design details are provided in the published protocol [44].

### Ethical Considerations

The University of Florida Institutional Review Board - mCHAMP (Mobile Childhood Health and Asthma Management Program) approved all study procedures (IRB201801092), and all participants provided informed consent. The participation window ranged from October 2018 to December 2019.

### System Development

The mCHAMP app targeted adult caregivers of children living with asthma and obesity between the ages of 6 and 12 years. A consumer-centered participatory design (CCPD) approach was used to identify user requirements, and summative usability testing was completed with caregivers to measure overall usability, learnability, and efficiency [46].

Using CCPD, we iteratively identified salient content and end user needs from the original CHAMP intervention and how to best translate that into the mCHAMP mobile app. Potential end users included family caregivers and nurses. Focus groups were the primary method of engagement with participants during initial system development. However, if participants were not able to attend one of the scheduled focus groups, individual interviews were conducted. The primary goal of these sessions was to identify which components of the CHAMP intervention resonated most with caregivers and the preferred format for those components [45]. Caregivers also described their families’ unique barriers to asthma and weight management. The use of CCPD allowed us to better understand the perspective of community members who might benefit from our intervention and allowed us to build the mCHAMP app in line with community needs.

We continued to engage caregivers, starting with formative usability testing and ending with summative testing. Formative testing uses qualitative approaches iteratively to identify and fix usability problems with small samples [47]. We engaged caregivers in 3 formative sessions, which provided feedback on the design of our initial prototype. Following these sessions, we developed an interactive mCHAMP prototype for iOS and Android users in collaboration with MEI Research, a nonprofit organization that specializes in the development of medical education content, including mHealth apps. The resulting prototype was evaluated through summative usability testing, which is the focus of this paper.

### Approach

Summative testing is used to evaluate a system’s usability and commonly includes metrics such as time on task, errors, user satisfaction, and user interface challenges [47]. Overall usability, or satisfaction, was assessed quantitatively using the Post-Study eHealth Usability Questionnaire (PSHUQ) and qualitatively through open-ended questions. To assess learnability, or ease of learning, we used time on task and errors. Efficiency was measured through the number of steps and overall time to complete a task.

### Setting and Recruitment

We recruited caregiver participants using purposive sampling from an outpatient pediatric pulmonary clinic and a hospital-affiliated integrated data repository that allows researchers to receive a list of patients who meet inclusion criteria and have consented to be contacted about research studies. Caregiver participants were parents or legal guardians of children who were aged 6 to 12 years, had a physician-verified diagnosis of asthma for at least 6 months before study participation, had a BMI at or above the 85th percentile, and lived with the caregiver participant. Participants were required to speak and read English. We excluded participants who had any significant cognitive impairment or developmental delay that would interfere with study task completion. We recruited RN participants from the pediatric pulmonary division. RN participant inclusion criteria were (1) providing care for children with asthma and (2) having worked in that capacity for at least 1 year. Due to institutional review board restrictions regarding storage of information on potentially eligible patients, we did not collect data on the total number of individuals contacted or response rates.

The summative usability target sample was 10 caregivers. Studies that include 10 participants in summative testing should find an average of 95% of system errors and a minimum of 82% of system errors [48]. In addition to caregivers, we targeted a sample size of 5 RNs for feedback on the final prototype.

### Procedures

**Figure 2:**
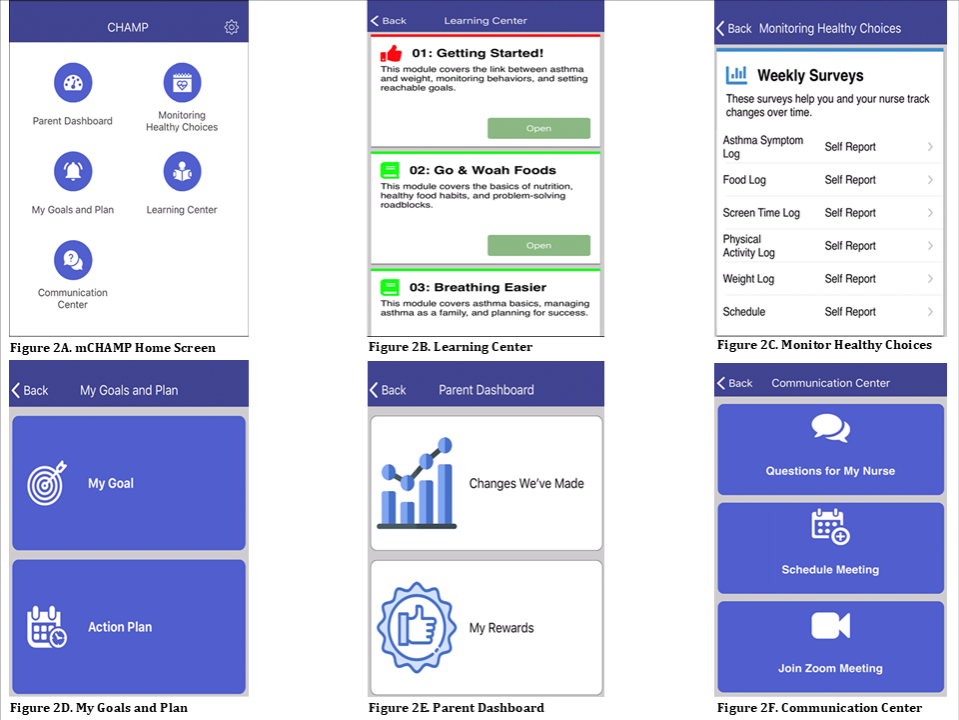
Mobile Childhood Asthma Management Program (mCHAMP)mCHAMP (A) home screen, (B) Learning Center, (C) Monitoring Healthy Choices, (D) My Goals & Plan, (E) Parent Dashboard, and (F) Communication Center.

The interactive mCHAMP prototype used in summative testing included six modules: (1) mCHAMP home screen, (2) Learning Center, (3) Monitoring Healthy Choices, (4) My Goals & Plan, (5) Parent Dashboard, and (6) Communication Center (Figure 2A-F). The home screen provides access to each section of the app. Within the Learning Center, users have access to general information about healthy eating, physical activity, and asthma symptoms in children aged 6 to 12 years. The Monitoring Healthy Choices section allows users to monitor their child’s screen time, eating, asthma symptoms, physical activity, and weight over time. In the My Goals & Plan section, we give users a place to set weekly goals. We ask whether they completed their goal, and if not, ask what got in the way. We also give users a place to view their plan and what to focus on during the upcoming weeks. This includes a list of action items related to their goals, the modules they are reviewing, and when they next meet with the nurse. In the Parent Dashboard, users can see how well they are managing their goals. This area includes rewards earned and how their child’s health has changed over time. The Communication Center provides a way for users to communicate directly with their nurse when they have questions about the program, their child’s asthma symptoms, or making healthy choices. We note that this feature should not be used for any emergency health issues or medication advice. Instead, this is a way for users to list questions for their next scheduled meeting with their nurse.

Summative usability testing sessions were conducted with a research team member and a caregiver. Each participant engaged with the mCHAMP app using a research mobile phone with an operating system that matched their current personal device’s operating system. We created a summative usability testing script that asked caregivers (n=10) to complete 15 total tasks across the 6 modules of the mCHAMP app. Study staff provided directed feedback only if participants were unable to complete a task that would impact subsequent tasks. Study staff asked participants to think aloud as they used the prototype and provided nondirective guidance when necessary. Screen recording captured participants’ on-screen movements. Following some tasks, open-ended questions were asked (eg, “Would you talk out loud about what this means to you?”) before prompting the next task. Following performance of all tasks, participants completed the PSHUQ [49].

### Nurse Feedback

While the mCHAMP app is primarily caregiver facing, it is intended to connect with RN interventionists. Therefore, in addition to obtaining caregiver feedback, we sought feedback from RNs as key stakeholders. Following summative testing with caregivers, a sample of 5 nurses received a brief overview of the project and a demonstration of the app’s functionality. Study staff (RJL and AF) conducted semistructured interviews with nurses. Questions were related to app content, the role of RNs in promoting patient self-management, and future implementation of a nurse-led intervention using this app in a pediatric pulmonology clinic or primary care setting.

### Measures

#### Demographics

Caregivers provided their age, gender, race or ethnicity, marital status, educational level, and household income. They also provided their children’s age and gender. RNs provided their gender, race or ethnicity, household income, and length of time working as an RN overall, in addition to time providing care to children with asthma.

#### Technology Use

Caregivers completed a brief technology use questionnaire adapted from the Health Information National Trends Survey to characterize how often they interacted with technology (eg, computers, cell phones, and tablets) and whether they used technology for health-related tasks or belonged to any social networking sites [50]. Information regarding general use of technology was collected to ensure that differences in usability metrics were not attributable to differences in general experience with technology as opposed to the mCHAMP app itself.

#### Usability

Overall usability, or satisfaction, was measured using the PSHUQ [49]. The PSHUQ comprises 18 items and uses a Likert scale ranging from 1 (“strongly agree”) to 7 (“strongly disagree”). Subscales include (1) system usefulness, which measures the ease of completing tasks; and (2) system quality, which measures satisfaction with the quality of the information and interface. We calculated the mean and SD for each of the individual items. We calculated subscale scores by averaging the items in each subscale. We averaged scores across all items to create an overall satisfaction score. Overall scores can range from 1 to 7, with lower scores indicating a higher degree of satisfaction.

#### Learnability

Learnability refers to the ability of a novice user to become proficient in a relatively short period [51]. Learnability metrics included number of hints and errors coded in each session. Hints were defined as any statement by the study staff that aided the caregiver in completing a task. Errors were defined as actions taken by participants that did not follow the expected steps in the script.

#### Efficiency

Efficiency is the average time it takes for users to complete a defined set of tasks [52]. Efficiency was measured through the total time to complete the tasks associated with a particular module and the number of individual steps taken to accomplish those tasks. Steps included the count of the following aspects: clicking (any click following the script), errors (any click that did not follow the script), error recovery (any click after the initial error to return to the task), entering text (any action involving the user typing into the app), and scrolling (any action taken to scroll through content on the app).

#### Analysis

Screen recordings were analyzed using NVivo (version 12; Lumivero) to describe and quantify usability measures. Recordings were coded using an a priori codebook that was developed by team members (RJL, KS, and YX) based on the usability testing script and planned usability metrics. After establishing intercoder reliability, 2 team members (KS and YX) coded these recordings using NVivo. Any disagreements on coding were resolved through discussion. Iterative content analysis was used for the open-ended questions and semistructured RN interviews.

## Results

### Caregiver Participants

#### Overview

Caregivers (n=10) were female (n=10, 100%) and mostly African American (n=8, 80%) and had an annual household income of less than US $25,000 (n=5, 50%; Table 1). Most of their children (n=6, 60%) were in the 99th BMI percentile. In terms of technology use, all caregivers (n=10, 100%) reported spending at least 2 hours per day engaging with their smartphone for activities other than phone calls, and half (n=5, 50%) reported 5 hours or more of daily use. All participants also reported belonging to a social networking site, and most engaged with digital health information (eg, websites and health-related apps).

**Table 1:** Caregiver demographics and technology use (n=10).

			Values
Caregiver age (y), mean (SD; range)			37.10 (6.76; 29-51)
Caregiver gender (female), n (%)			10 (100)
**Caregiver race, n (%)**			
	African American/Black	8 (80)	
	Hispanic/Latino White	1 (10)	
	Non-Hispanic/non-Latino White	1 (10)	
**Caregiver marital status, n (%)**			
	Married	2 (20)	
	Divorced	2 (20)	
	Single	6 (60)	
**Caregiver educational level, n (%)**			
	Did not graduate high school	1 (10)	
	Partial college or specialized training	5 (50)	
	College graduate	3 (30)	
	Graduate or professional degree	1 (10)	
**Annual household income (US $), n (%)**			
	<5000	2 (20)	
	5000-11,999	1 (10)	
	12,000-15,999	1 (10)	
	16,000-24,999	1 (10)	
	25,000-34,999	2 (20)	
	35,000-49,999	2 (20)	
	50,000-74,999	1 (10)	
Child age (y), mean (SD; range)			10.10 (1.73; 8-12)
Child BMI percentile, mean (SD; range)			97.20 (2.78; 92-99)
**Caregiver cell phone use on a typical day within the previous month (excluding time spent talking on the phone; h), n (%)**			
	2	2 (20)	
	4	3 (30)	
	≥5	5 (50)	
**Caregiver computer use on a typical day within the previous month (excluding work-related use; h), n (%)**			
	<1	2 (20)	
	1	2 (20)	
	2	3 (30)	
	3	3 (30)	
**Caregiver tablet use on a typical day within the previous month (h), n (%)**			
	<1	2 (20)	
	2	2 (20)	
	≥5	1 (10)	
	Did not use a tablet	5 (50)	
**Caregiver technology use within the previous year, n (%)**			
	Belonged to social networking site	10 (100)	
	Used the internet to look up health or medical information	8 (80)	
	Used mobile health apps on their phone or tablet	9 (90)	
	Used email, the internet, or an app to communicate with a physician or physician’s office	7 (70)	
	Participated in an online support group for people with similar health or medical issues	1 (10)	

#### Summative Usability

Participants’ scores on the PSHUQ indicated high overall satisfaction, with a mean rating of 1.13 (SD 0.24) and possible scores ranging between 1 (highest) and 7 (lowest; median 1, range 1-1.67). Caregivers also reported high levels of usefulness (mean 1.08, SD 0.10; median 1.00, range 1.00-1.20) and quality (mean 1.16, SD 0.33; median 1.00, range 1.00-1.90).

#### Learnability

Most participants (n=7, 70%) were able to complete all 15 tasks across the 6 modules with 2 or fewer hints (Table 2). Overall, participants received 0.53 hints per module on average (SD 0.55; range 0-1.8) and committed an average of 0.38 (SD 0.42; range 0-0.67) errors per module. One data point for hints represented an outlier with a total of 11 hints over the 6 modules and an average of 1.83 hints per module. Data for errors did not include any outliers. All participants were able to complete module 1 (mCHAMP home screen) without hints or errors. Most (n=9, 90%) were able to complete the 5 tasks in the Learning Center with 1 or no hints. Participants were also able to complete the Monitoring Healthy Choices tasks with 1 or no errors (n=8, 80%) and 1 or no hints (n=9, 90%). Module 4, My Goals & Plan, was easily navigated by participants, with nearly all (n=9, 90%) not receiving any hints and only 10% (n=1) of the participants committing 1 error. The 2 tasks in module 5, Parent Dashboard, were more challenging, with half of the users receiving 2 or more hints (mean 1.2, SD 1.4; range 0-3). Users were able to use the Communication Center easily, with 70% (n=7) not requiring any hints; the remining 30% (n=3) required 2 hints each, and only 20% (n=2) of the users committed 1 error each.

**Table 2:** Learnability and efficiency scores.

Module	Learnability, mean (SD; range		Efficiency, mean (SD; range	
	Hints	Errors	Steps	Time (min)
1. mCHAMP home screen	0 (0; 0)	0 (0; 0)	1 (0; 1-1)	0.1 (0.1; 0.0-0.2)
2. Learning Center	0.6 (1; 0-3)	0.6 (0.8; 0-2)	16.4 (2.6; 11-19)	6.6 (1.8; 3.1-8.6)
3. Monitoring Healthy Choices	0.06 (0.7; 0-2)	0.5 (0.8; 0-2)	20.4 (8.3; 13-34)	3.1 (1; 2-5)
4. My Goals & Plan	0.2 (0.6; 0-2)	0.1 (0.3; 0-1)	14.2 (2; 10-17)	3.2 (1.2; 1.4-4.6)
5. Parent Dashboard	1.2 (1.4; 0-4)	0.9 (1.1; 0-3)	16.8 (2.5; 14-23)	1.6 (0.7; 0.9-2.7)
6. Communication Center	0.6 (1; 0-2)	0.2 (0.4; 0-1)	16 (3.6; 9-21)	3.2 (1.7; 1.2-2.3)

#### Efficiency

The average total time to complete all tasks across the 6 mCHAMP modules was 17 (SD 3.9; range 11.4-24.1) minutes. Users spent the most time on the Learning Center (mean 6.6, SD 1.8; range 3.1-8.6 minutes), which included 2 videos totaling 1.5 minutes. Users completed module 1, the home screen, most quickly (mean 0.1, SD 0.04; range 0.02-0.17 minutes), followed by module 5, taking an average of 1.6 (SD 0.69; range 0.9-2.72) minutes to view the graphs and access the rewards. Modules 3, 4, and 6 took between 2.49 and 3.23 minutes on average. Table 2 provides efficiency data.

### User Preferences

#### Electronic (Audio or Visual) Media

Participants felt that the videos were a good length (less than 1 minute each). They also liked the use of color and felt that the animation made them enjoyable and inviting to children and caregivers. When asked whether they preferred one video over the other, nearly half (n=4, 40%) of the participants talked about the importance of matching the gender of the character to the child watching, and 10% (n=1) also commented that it was important to depict racial diversity. A few participants (n=3, 30%) preferred the video that gave actionable information about activity and included more peer interactions. However, 10% (n=1) of the participants noted that they preferred the video that depicted how asthma can be isolating to a child because they felt that it was important for children to know that their experience is common. That participant also liked how that video included the experience of the child and the caregiver.

When asked about video content, caregivers were generally positive (n=9, 90%), with only 10% (n=1) stating that it was not valuable. That participant stated a preference for engaging directly with the nurse rather than with videos. Some participants (n=2, 20%) felt that it was important for content to be engaging for both children and adults, whereas 10% (n=1) of the caregivers stated that they would pass the app to their children and say, “Here you go. Mommy got this. You might wanna read it. You might wanna listen at it. It’s cute” [U18].

However, there was a strong preference (n=9, 90%) for child-facing content. Participants stated that information about activity levels was particularly important (n=5, 50%), with 20% (n=2) stating that they did not know that being active was important for asthma control and 10% (n=1) stating that they sometimes struggled to encourage activity even though they knew that it was the right thing to do. One participant would have liked additional information on identifying the warning signs of an asthma attack. The videos may also meet the needs of health care providers, with one caregiver stating that her physician wished she had a child-friendly video to explain what happens in the lungs during an asthma attack at their last appointment.

#### Static (Text) Media

Regarding written content, most caregivers (n=7, 70%) were comfortable and wanted the information in a static form but wanted an alternative form of media to support flexibility and choice regarding how to consume the content. Caregivers overwhelmingly supported the idea of having either videos or a “read aloud” audio function (n=10, 100%).

#### Caregiver Engagement

When asked about the survey task, caregivers reported it being easy to complete (n=9, 90%) and easy to navigate (n=2, 20%). One participant did voice concerns about how accurate they could be regarding entering information as their child spent so much time at school and in other activities where they did not see food or activity choices. There was also one comment about the use of the label “self-report” and that this phrase might be jargon; the use of more plain language for each task (eg, “food log”) would be better.

Features that participants discussed in the reward section included a preference for tracking progress toward goals or visibility of status (n=4, 40%), animated congratulations when awards were unlocked (n=4, 40%), streak tracking to encourage consecutive goal achievement (n=3, 30%), and tailored notifications with actionable ideas when goals had not yet been achieved (n=2, 20%). The importance of visibility of status was described by one user as follows:

Okay, I did it without my asthma gettin’ in the way. It’d convince him to keep goin’ forward instead of stoppin’ or not wantin’ to do it because of his asthma.

U16

When asked about the reward center, many users (n=6, 60%) reiterated the desire for child-facing content. Many (n=5, 50%) talked about the value of having both child-facing content and caregiver-facing content. This was sometimes described as having one account per family, with the ability to log in on multiple devices and select either a child or caregiver role. When parent-facing content was discussed, the use of minimalist design using numbers rather than stickers or icons was described (n=2, 20%), and more icons or visual depictions were preferred for child-facing content (n=3, 30%). One user would have liked to see additional nutrition-related content in this area, and one user suggested adding interactive game content to help teach health promotion principles.

The concept of person-centered support was also observed in the preference for tailored notifications, with one user stating the following:

Yeah, like at the end of the week, when a child hasn’t met their goals, it’s okay to not always succeed, as long as you keep trying, or try, try, try again. You get knocked down, try again. Tomorrow’s a new day. There’s a thousand statements that you could put in there. Just some kind of a support.

U17

One participant valued the automated integration of Fitbit data, and another commented on the importance of a mobile app for in-the-moment data entry for logging symptoms. One participant also stated that she would have liked a way to interact with other caregivers (eg, peer support).

#### Health Care Provider Engagement

When asked about the feature to connect with a nurse, most (n=8, 80%) liked the ability to collaborate with the nurse, with some (n=3, 30%) stating that this feature might improve their access to care. The video component to the nurse communication was important to at least one caregiver, and 20% (n=2) commented about the need to have a specialized nurse knowledgeable about nutrition. The only difficulty discussed regarding the Communication Center was in navigating back to the main content after speaking with the nurse via Zoom.

### RN Feedback

Semistructured interviews with 5 nurses who provided care to pediatric patients with asthma were completed. Nurses were recruited from diverse settings, including primary care, specialty care, and clinics serving patients with varying socioeconomic statuses. Inclusion of diverse participants allows for insight into potential need and important contextual factors in diverse settings for future implementation studies.

All 5 nurses strongly endorsed the role of the nurse to include education and promotion of self-management (self-efficacy). For example, nurses indicated that “Oh, yeah, that’s our core function” and “that’s what nursing does. Nursing is the education point of healthcare.” Nurses all endorsed that mCHAMP had necessary activity-related content. One felt that there was room for improvement with the nutritional content. Several nurses also expressed concerns regarding low levels of literacy in the target population. This was further supported by nurses recommending additional video and pictorial content. Nurses liked that both genders were represented in the videos currently integrated into mCHAMP. Nurses also responded favorably to the Parent Dashboard and Communication Center content. They particularly liked the ability to see trends in the caregiver dashboard and the use of video in the Communication Center to enable some visual assessment compared to a phone-only interaction. They liked the use of in situ patient-entered data and the ability to monitor pertinent data in between appointments.

The use of mCHAMP in primary care was endorsed by all but one nurse (4/5, 80%), who felt that she did not have enough knowledge of the primary care environment to provide an informed response. Most participants also supported the use of mCHAMP within specialty care, and some proposed the use of a centralized nurse with specialized asthma experience to enable smaller clinics to participate in the program. Several nurses also stated that integration of mCHAMP data into the electronic health record (EHR) would be important to the feasibility and sustainability of the intervention. Nurses also talked about the importance of integrating the intervention into existing workflows to promote health care provider buy-in. To achieve integration of data within the EHR, a health care provider dashboard was recommended.

## Discussion

### Principal Findings

The overarching goal of this study was to conduct summative usability testing of the mCHAMP app with caregivers and nurses. Overall, the mCHAMP app was viewed positively by caregivers, as evidenced by receiving high ratings for satisfaction, perceived system usefulness, and quality on the PSHUQ. Our results are comparable to findings from other health technology usability studies. Sheehan and Lucero [53] reported similarly favorable PSHUQ scores (mean 1.58; median 1.38) for their Self-Assessment via a Personal Health Record fall prevention system among older adults, demonstrating high satisfaction with system usefulness (mean 1.84) and quality (mean 1.43), with possible scores ranging between 1 (highest) and 7 (lowest). Both studies show that users found these health self-management systems easy to use and of high quality, with our caregivers rating the mCHAMP app slightly more favorably on usefulness (mean 1.08, SD 0.10) compared to the Self-Assessment via a Personal Health Record study. These comparable satisfaction scores across different health domains and user populations suggest that health applications can achieve high user acceptance when developed using well-designed, theory-driven usability frameworks.

Furthermore, our metrics of learnability and efficiency for the mCHAMP app with caregivers were favorable. Caregivers were able to complete our list of prespecified module tasks with few hints, and the average time spent on each module of the app ranged from under 1 minute to just over 6 minutes. Consistent with our usability data, qualitative feedback provided by caregivers during the think-aloud testing indicated that they were pleased with several mCHAMP app features, including the animated videos and ease of completing self-report surveys. Caregivers expressed a desire for an intervention that was easy to use and could integrate into their busy family lives during requirement gathering sessions that we conducted when designing the mCHAMP app. We appear to have met this expectation based on the usability, learnability, and efficiency data from this study.

A main point of innovation for the mCHAMP app is that it leverages the expertise and training of RNs as interventionists to develop caregiver self-management skills. An important finding from our usability testing was that caregivers were indeed enthusiastic about working with an RN interventionist to facilitate their use and uptake of self-management skills via the mCHAMP app. Caregivers stated they liked having the ability to reach out via videoconference to the RN interventionist rather than googling or calling as it would give them the ability to show the RN what was going on rather than being limited to a description over the phone. Previous research has demonstrated that technology-based interventions that incorporate RNs as care deliverers are feasible and efficacious at reducing morbidity in pediatric asthma [54,55]. Given the ubiquity of mobile phones, and in the context of a health care system that emphasizes care within a medical home model of care [56], the use of RNs as interventionists in the mCHAMP app to concurrently target asthma and obesity extends prior work and generates confidence that there is a viable framework for scalability.

Given the central importance of self-monitoring in the self-management of chronic illness, we designed the mCHAMP app to enable caregivers to enter data (eg, asthma symptoms, food logs, and screen time) and synchronize with a Fitbit to facilitate tracking of important health behaviors and progress toward self-management goals [57,58]. Caregivers responded positively to these components of the mCHAMP app and had little difficulty completing surveys during testing. Moreover, a general theme of feedback from caregivers was that they valued how their children’s health data could subsequently lead to a more personalized intervention experience. Examples given by caregivers included informing more meaningful interactions with the RN interventionists and that having their children’s health data in a central location could increase their accuracy when attending appointments with their children’s physicians. We posit that these data, if regularly accessed by the caregiver, could also help strengthen connections between health behaviors (eg, physical activity levels and food intake) and important outcomes (eg, asthma symptoms and weight) in children with comorbid asthma and obesity.

Caregivers noted several areas in which we could make further improvements to the mCHAMP app. Specifically, caregivers wanted flexibility to engage with educational content on the app in multiple ways, including the addition of more videos and read-aloud functions to the current prototype. Notably, while using the Parent Dashboard module, participants required more hints compared with other sections of the mCHAMP app. Our review of the data indicated that most hints were required following the task prompt “Show me how you would access the graphs that show changes in your activity and your child’s activity over time,” with more than half of the participants requiring at least one hint before selecting the Parent Dashboard. Common incorrect selections by caregivers included them selecting the Learning Center, Monitoring Healthy Choices, or Communication Center modules. The range of actions taken by caregivers suggests that we should refine the mCHAMP app to better differentiate where visual health data are stored to improve usability.

Many caregivers indicated that they wanted the mCHAMP app to include child-focused content. We considered including child-focused content during our initial development of the mCHAMP app but decided to focus on caregivers due to the developmental level of our target sample (aged 6-12 years) and based on previous studies that have demonstrated that in-person caregiver BFIs are as efficacious as those that include caregiver-child dyads [24]. In addition, the emerging data on the added benefit of including children in eHealth or mHealth interventions for child weight management are mixed. However, recent data suggest that including children as active recipients of a technology-based intervention is associated with increased efficacy [59]. Thus, an important direction for our future work in this area is to identify developmentally appropriate ways to include children in mCHAMP.

RNs also had a favorable response to the mCHAMP app. They valued the use of caregiver-entered health data, which is consistent with the literature on the benefits of patient-reported outcomes. Systematic use of patient-reported data in between care may support patient-provider communication in addition to fostering clinician awareness of health status in between episodes of care [60]. RNs also recommended collecting data on medication use to provide information that may be important for asthma management. This recommendation is also supported by literature on the use of patient-entered data to facilitate medication adherence among patients with chronic diseases [61]. Finally, RNs also had positive feedback regarding the videoconferencing features of the mCHAMP app as a mechanism to provide support between intervention sessions.

Nurses raised important contextual factors, such as the ability to integrate mCHAMP into the EHR and potentially differing workflows among participating clinics as concerns related to future implementation. These concerns could be mitigated through the use of workflow analysis in future studies and the use of interoperability standards to enable integration of data into the EHR.

### Limitations

While our usability evaluation of the mCHAMP app was strengthened by the inclusion of the perspectives of potential end users or family caregivers and health care providers, there are evaluation weaknesses overall that should be addressed in future research. First, we observed that all the usability evaluations were conducted using the iOS operating system based on family caregivers’ actual mobile phone type. Second, future research should include additional family caregivers whose children are affected by obesity and asthma that were not included in our study, namely, Hispanic English- and Spanish-speaking caregivers. Third, while our sample size of 10 caregivers was adequate for identifying usability issues, this limits our ability to fully understand the range of experiences and preferences across diverse populations of caregivers managing childhood asthma and obesity.

The homogeneous nature of our sample may not capture usability challenges that could emerge among caregivers with different technological comfort levels or cultural backgrounds. Only 40% of child patients with asthma are coded as Black or African American in the data repository, which does not contain information on obesity. Moreover, without demographic data on caregiver characteristics in the broader repository population, we cannot fully assess the extent of these potential biases. In addition, there are opportunities to engage family caregivers from varied geographic locations and settings, including primary care in urban and suburban clinics with health care providers whose experience may be different from that of health care providers in specialized pulmonary clinics.

### Conclusions

Our study was able to identify key components of the original CHAMP intervention to develop and test the usability of the mCHAMP app by engaging stakeholders using the CCPD process. Caregivers and nurses had favorable feedback on mCHAMP. With some minimal revisions, the mCHAMP app could be used in future pilot-testing. This will enable the research team to test the hypothesis that translating CHAMP from an in-person intervention to an mHealth intervention will improve retention rates while maintaining high levels of satisfaction. The mCHAMP app has the potential to increase self-management for pediatric patients with asthma dealing with multimorbidity, which could translate into improved patient outcomes and long-term benefits for health systems. The use of mCHAMP may also enable novel clinical outcome studies based on patient-reported data from the app.

## Abbreviations

BFI: behavioral family lifestyle intervention
CCPD: consumer-centered participatory design
CHAMP: Childhood Asthma Management Program
EHR: electronic health record
mCHAMP: Mobile Childhood Asthma Management Program
mHealth: mobile health
PSHUQ: Post-Study eHealth Usability Questionnaire
RN: registered nurse

## Data Availability

The datasets generated or analyzed during this study are available from the corresponding author on reasonable request.
